# Supplementary material for: Evidence for Biological Age Acceleration and Telomere Shortening in COVID-19 Survivors
Source: Int J Mol Sci. 2021 Jun 7;22(11):6151. doi: 10.3390/ijms22116151 (PMC8201243; doi:10.3390/ijms22116151)
Supplement: Supplementary file 1 [file ijms-22-06151-s001.zip › ijms-1240093-supplementary.pdf]

Supplementary.

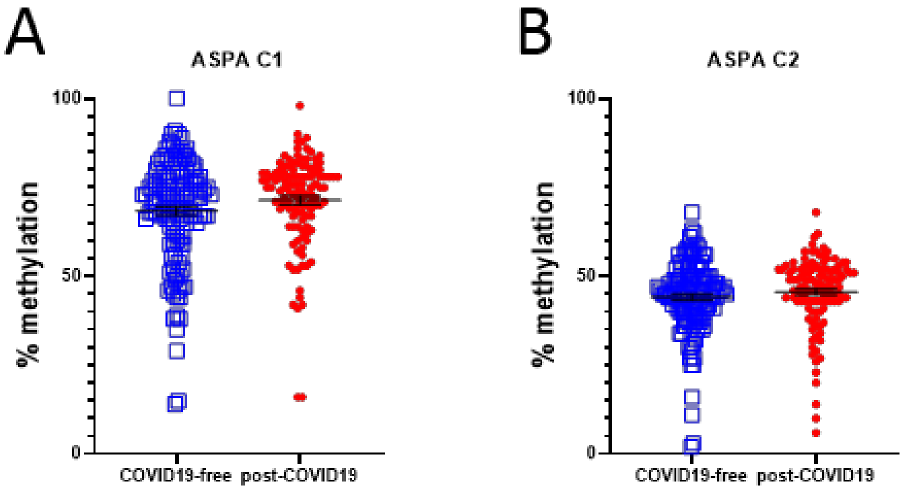

Supplemental Figure S1: CpGs methylation level in ASPA target sequence. No differences observed between COVID19-free and post-COVID19.

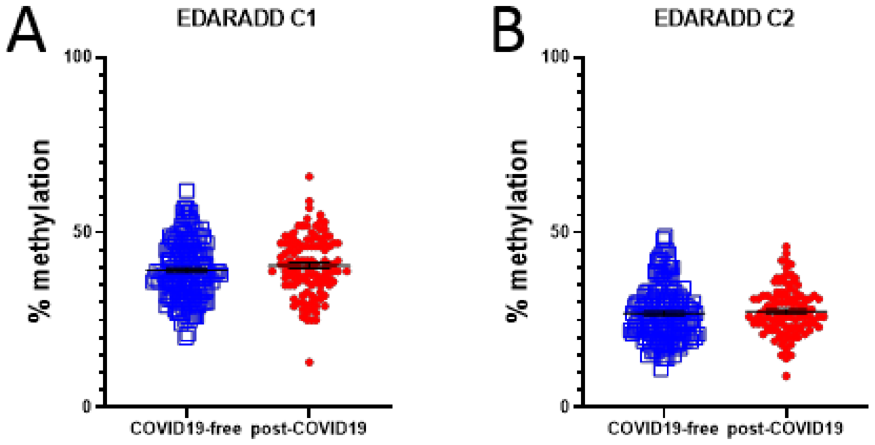

Supplemental Figure S2: CpGs methylation level in EDARADD target sequence. No differences observed between COVID19-free and post-COVID19.

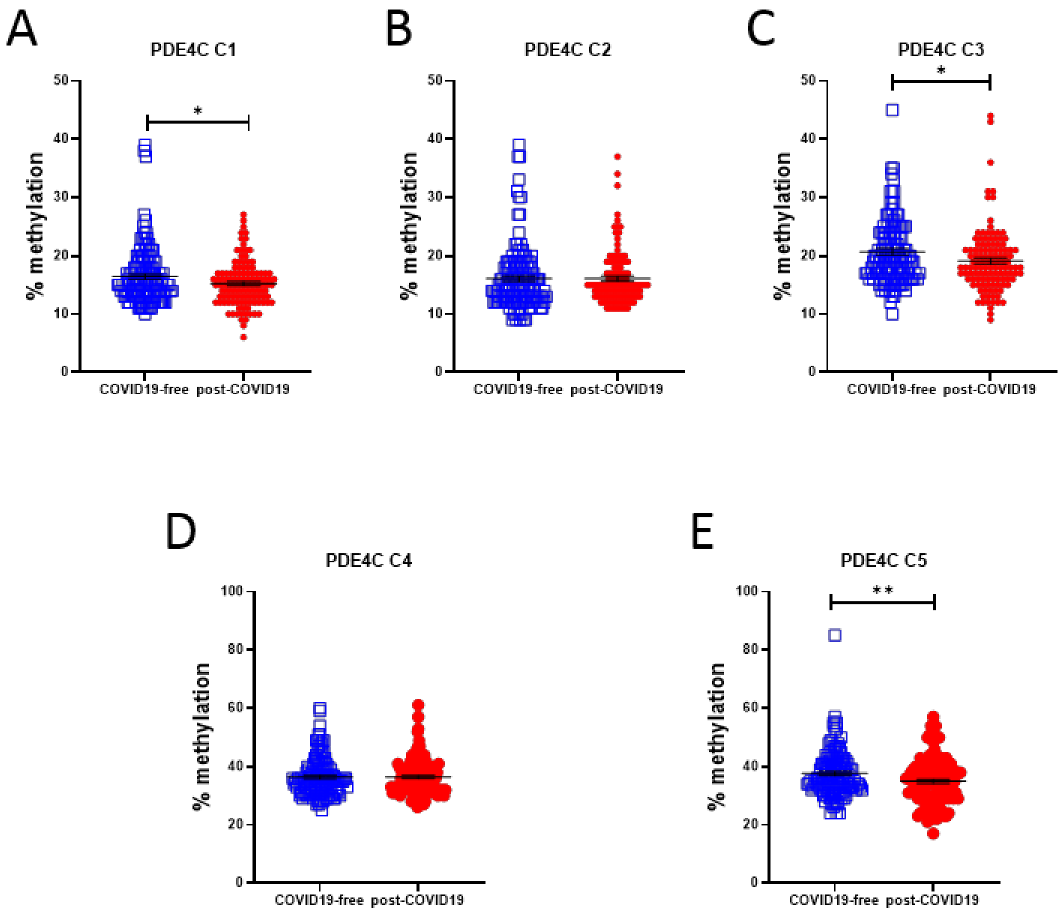

Supplemental Figure S3: CpGs methylation level in PDE4C target sequence. C1, C3 and C5 show a significant increase of methylation in COVID19-free compared to COVID19 survivors. C1 p-value amounts to 0.0313; C3 p-value is 0.0356; C5 p-value is 0.0085

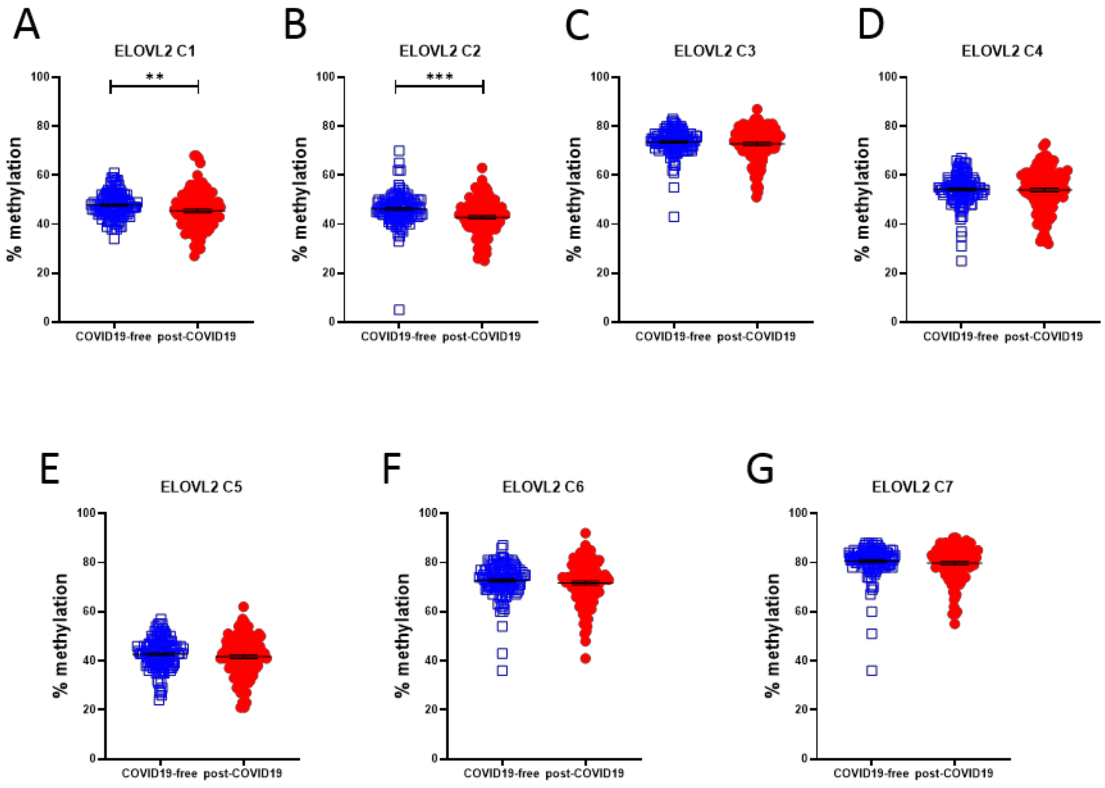

Supplemental Figure S4: CpGs methylation level in ELOVL2 target sequence. Differences are observed in C1 and C2

Supplemental Table S1: primer used for PCR and pyrosequencing (ELOVL2, ASPA, PDE4C and EDARADD) and primer designed for qPCR.

gene	primer	Modification 5’	sequence
ELOVL2	forward	Biotin	AGGGGAGTAGGGTAAGTGAGG
	reverse		AACAAAACCATTTCCCCCTAATAT
	sequencing		ACAACCAATAAATATTCCTAAAACT
ASPA	forward		TGTTGAAGAATATATATAAAAGGTTGT
	reverse	Biotin	ATCTTACCCAAAATTTTCAAAATCAAA
	sequencing		TGAAGAATATATATAAAAGGTTGTT
PDE4C	forward		GTAGGAGGAAAAGGGTTAGGAGAG
	reverse	Biotin	CCCAAACCCCTTTCTCTAAC
	sequencing		GAATAGAAGAGTTGTTGGATG
EDARADD	forward		GGAGTTTGTTATGGAAGAAGTAATAG

	reverse	Biotin	ATCCTCCCACCTACAAATTC
	sequencing		TGTTATGGAAGAAGTAATAGA
GAPDH	forward		GTCTCCTCTGACTTCAACAGCG
	reverse		ACCACCCTGTTGCTGTAGCCAA
ACE2	forward		GGACCCAGGAAATGTTCAGA
	reverse		GGCTGCAGAAAGTGACATGA
DPP4	forward		CAAATTGAAGCAGCCAGACA
	reverse		CACACTTGAACACGCCACTT

Supplemental Table S2: target sequences set for pyrosequencing. In bold are the CpG used to calculate the DNAmAge.

**Target gene**	**Sequence to analyse**
**ELOVL2**	CCRTAAACRTTAAACCRCCRCRC**R**AAACCRAC
**ASPA**	ATTTTTGGAGGAATTTATGGGAATGAGTTAATYGGAGTATTTTTGGTTAAGTATTGGTTAGAGAATGG**Y**GTTGAGAT
**PDE4C**	**Y**GGATGGGGYGTYGGGGTTGTYGTTATAGGTGTTTYGGGGTTTT
**EDARADD**	TTG**Y**GAGAAGATGTTYGTTGG

Supplemental Table S3: chromosomal localization of age-related CpGs used to calculate the DNAmAge.

**CpG Analysed on target sequence**	**Localization on GRCh38/hg38**
**ASPA (CpG2)**	chr17:3476273 - sense
**EDARADD(CpG1)**	chr1:236394383- antisense
**ELOVL2 (CpG6)**	chr6:11044656 - antisense
**PDE4C (CpG1)**	Chr19:18233070- sense
